# When precaution outlasts evidence: reconsidering blood collection in French Guiana

**DOI:** 10.1016/j.lana.2026.101604

**Published:** 2026-08-21

**Authors:** Loïc Epelboin, Mathieu Nacher, Félix Djossou, Olivier Hermine

**Affiliations:** a Unité des Maladies Infectieuses et Tropicales, Centre Hospitalier Universitaire de Guyane, Cayenne, 97300, French Guiana, France; b CIC INSERM 1424, Centre Hospitalier Universitaire de Guyane, Cayenne, 97300, French Guiana, France; c UA17 Santé des Populations en Amazonie, INSERM, Cayenne, 97300, French Guiana, France; d CEREMAST, Imagine Institute, INSERM U1163, Paris, France; e Department of Hematology, Necker-Children's Hospital (AP-HP5), Paris-Centre University, Imagine Institute, Paris, France; f Paris-Centre University, Imagine Institute, Laboratory of Molecular Mechanisms of Hematologic Disorders and Therapeutic Implications, INSERM UMR 1163, Paris, France

France has suspended blood collection in French Guiana, a French overseas territory in South America since 2005 because of concerns regarding transfusion-transmitted infections, particularly *Trypanosoma cruzi*, HIV, malaria, and other tropical pathogens. At that time, the precautionary principle was understandable. Diagnostic tools were less sensitive, the epidemiology of several infections remained poorly characterized, and the decision followed the HIV-contaminated blood crisis that profoundly shaped French transfusion policy. Twenty years later, however, both the epidemiological context and transfusion medicine have changed substantially, whereas the territorial suspension remains unchanged.^1^, ^2^, ^3^, ^4^

Current evidence no longer supports considering French Guiana as presenting an unmanageable transfusion risk. Chagas disease, which became the principal justification for maintaining the suspension, is now considered as uncommon, with an estimated prevalence of only 0·17% and no documented cases of transfusion-transmitted infection in the territory.^3^ HIV management has also changed dramatically through earlier diagnosis, widespread antiretroviral treatment, and routine nucleic acid testing, substantially reducing residual transfusion risk.^4^ Similarly, improved serological and molecular testing now enables more reliable HTLV-1&2 screening than two decades ago. Likewise, although malaria has recently resurged because of *Plasmodium vivax*, transmission is largely restricted to well-defined populations living or working in remote forested areas and can be addressed through targeted donor selection and highly sensitive molecular screening.^5^ French Guiana has repeatedly experienced outbreaks of arboviruses such as dengue, chikungunya, and Zika virus over the past two decades and is currently facing a new chikungunya epidemic.^6^^,^^7^ In addition, endemic or sporadic circulation of Alphaviruses (Mayaro and Tonate viruses), is regularly documented, and Orthobunyaviruses such as Oropouche virus infections have recently been identified. However, molecular diagnostics and blood safety technologies have advanced considerably since the early 2000s. Similar epidemiological situations are now successfully managed elsewhere through temporary donor deferral, nucleic acid testing, and epidemiology-driven molecular screening rather than permanent suspension of blood collection.

Whether blood collection in French Guiana can be made safe is no longer a question. So, is a territory-wide suspension proportionate to the current level of risk?

This issue is particularly important because French Guiana has major unmet transfusion needs. The territory has one of the highest incidences of sickle cell disease within the French healthcare system and a population characterized by exceptional immunohematological diversity.^8^ Afro-descendant communities, Indigenous Amerindian peoples, and Businenge (Maroon) populations carry blood group phenotypes and Rhesus (RH) variants that are underrepresented in mainland France but are essential for providing compatible blood products to patients with sickle cell disease and complex alloimmunization profiles.^9^ Continuing to exclude these populations from blood donation not only limits local transfusion capacity but also deprives the national blood system of strategically important donors.

Importantly, this reassessment is also supported by national expert bodies. In 2025, the French High Council for Public Health concluded that blood collection could safely resume in French Guiana provided that enhanced donor selection, systematic screening for *T*. *cruzi*, HTLV-1&2, and malaria, and epidemiology-driven management of arboviral threats were implemented.^10^ These recommendations mirror the evolution of transfusion practice towards individualized risk assessment rather than broad geographic exclusion.

Restarting blood collection should nevertheless not simply restore the system that existed before 2005. It provides an opportunity to establish a modern programme integrating molecular diagnostics, prospective haemovigilance, and targeted recruitment of donors carrying rare blood phenotypes. It would also create opportunities for epidemiological surveillance, transfusion research, and studies exploring donor acceptability in a multicultural territory where Indigenous communities have historically been absent from blood donation programmes.

French Guiana illustrates a broader challenge in public health: precautionary measures are often easy to introduce but difficult to revisit once scientific evidence evolves. Two decades after the suspension of blood collection, the available epidemiological data, advances in transfusion safety, and recent national expert recommendations all support replacing a territory-wide exclusion with a modern, evidence-based strategy founded on individualized risk assessment and continuous surveillance. Beyond transfusion medicine, resuming blood collection would contribute to the ongoing strengthening of the health system in French Guiana, illustrated by the creation of the Universitary Hospital of French Guiana in 2025, and support efforts to reduce long-standing territorial health inequalities.

## Contributors

Loïc Epelboin conceived the manuscript and drafted the first version. Mathieu Nacher, Félix Djossou, and Olivier Hermine contributed to the conceptualization of the manuscript, critically revised the text, and approved the final version.

## Declaration of interests

The authors declare no conflicts of interest.
